# EMF Monitoring—Concepts, Activities, Gaps and Options

**DOI:** 10.3390/ijerph110909460

**Published:** 2014-09-11

**Authors:** Gregor Dürrenberger, Jürg Fröhlich, Martin Röösli, Mats-Olof Mattsson

**Affiliations:** 1 Swiss Research Foundation for Electricity and Mobile Communication, c/o Eidgenössische Technische Hochschule Zürich (ETH Zürich), Gloriastrasse 35, 8092 Zurich, Switzerland; 2 Institute for Electromagnetic Fields, Eidgenössische Technische Hochschule Zürich (ETH Zürich), Gloriastrasse 35, 8092 Zurich, Switzerland; E-Mail: j.froehlich@ifh.ee.ethz.ch; 3 Swiss Tropical and Public Health Institute (Swiss TPH), Socinstrasse 59, Postfach, 4002 Basel, Switzerland; E-Mail: martin.roosli@unibas.ch; 4 University of Basel, 4002 Basel, Switzerland; 5 Austrian Institute of Technology (AIT), Konrad-Lorenz-Strasse 24, 3430 Tulln, Austria; E-Mail: mats-olof.mattsson@ait.ac.at

**Keywords:** electromagnetic fields, exposure monitoring, exposure metrics, exposure assessment, monitoring paradigm, personal exposure, exposure policy, epidemiology, public health policy

## Abstract

Exposure to electromagnetic fields (EMF) is a cause of concern for many people. The topic will likely remain for the foreseeable future on the scientific and political agenda, since emissions continue to change in characteristics and levels due to new infrastructure deployments, smart environments and novel wireless devices. Until now, systematic and coordinated efforts to monitor EMF exposure are rare. Furthermore, virtually nothing is known about personal exposure levels. This lack of knowledge is detrimental for any evidence-based risk, exposure and health policy, management and communication. The main objective of the paper is to review the current state of EMF exposure monitoring activities in Europe, to comment on the scientific challenges and deficiencies, and to describe appropriate strategies and tools for EMF exposure assessment and monitoring to be used to support epidemiological health research and to help policy makers, administrators, industry and consumer representatives to base their decisions and communication activities on facts and data.

## 1. Introduction

Public exposure to electromagnetic fields (EMF) is continuously changing in the two main frequency domains, *i.e.* radiofrequency (RF; 100 kHz–300 GHz) and extremely low frequency (ELF; 0 Hz–300 Hz), due to new infrastructure deployments (4th generation mobile phone networks, smart grids for efficient electricity distribution), smart environments (small-scale wireless sensors, monitoring and access networks), and new wireless consumer devices. Furthermore, exposure from applications in the intermediate frequency (IF; 300 Hz–100 kHz) and the terahertz frequency (TF; >300 GHz) domains will become more prominent in the future [1,2].

Against this background, crucial deficits in current EMF exposure assessment and monitoring have to be overcome. The key deficit relates to the determination of personal exposure levels. Little reliable data about personal exposure levels and patterns is available, and nothing is known about (potential) lifetime exposure of young people. This lack of knowledge increases public concerns about electromagnetic exposure and potential health risks [3,4], and impedes effective exposure policies including appropriate risk communication. 

Apparently, the lack of monitoring data creates even among experts quite unrealistic perceptions about the EMF exposure of the population. A recent systematic evaluation of European ELF-EMF measurement studies concluded that median exposure is about 0.02 µT and only about 5% of the population is exposed above 0.1 µT [5]. In contrast, according to average exposure ratings done by 39 European experts, roughly 50% of the population would be exposed above 0.1 µT and about 5% above 2 µT (supplementary material B in [5]).

Regarding exposure policy, protection limits have been suggested by international bodies like the International Commission on Non-Ionizing Radio Protection (ICNIRP) [6,7] or the International Commission on Electromagnetic Safety (ICES) [8]. These guidelines protect people from known health effects with a substantial safety margin (often 50 for the public and 10 for occupationally exposed people). The fundamental limits (called basic restrictions) refer to the biological effects induced by incident electromagnetic fields. In the RF range, the relevant quantity of the basic restriction is the SAR (see next section). In the low frequency and intermediate frequency domain the induced electric field strength in human tissues is quantified. In the terahertz range power density is defined as the basic quantity. In 1999, the EU established a common protective framework with a recommendation on the limitation of the exposure of the general public to electromagnetic fields [9]. Moreover, in Europe, telecommunications equipment should comply with the RTTE directive which requires that products comply with the European Council recommendation [10].

Regarding public concerns: In spite of above mentioned protection limits and regulations, there are still considerable public concerns about possible health effects induced by EMF, as indicated by the EUROBAROMETER 2010 survey [11]. There is also considerable public confusion and misunderstandings regarding the ratio and magnitude of the electromagnetic fields within the different bands as well as the qualitative differences between various sources, for instance close-to-body devices and infrastructure installations, and their contribution to the total exposure. To respond to these concerns improved exposure assessment methods and monitoring concepts that generate valid data about real personal (and average population) exposures in harmonized campaigns have to be developed and implemented.

In a nutshell: without knowledge about real exposures, health risk assessments cannot be carried out, policymakers cannot establish evidence-based management measures and effective health risk communication programs, and industries cannot anticipate neither potential exposure impacts of new technologies nor potential regulatory developments, entailing, for instance, delays in the growth of new technology-markets. From this overall perspective, the following scientific challenges need to be overcome:

- Collection of systematic data and establishment of a paradigm to monitor EMF exposure;
- Development of appropriate equipment to assess and monitor personal EMF exposures;
- Development of appropriate equipment and data interpretation standards for near-field sources (devices used close to the body) in particular;
- Development of reliable exposure assessment methods tailored to the needs of epidemiological studies;
- Reduction of the large uncertainties in EMF exposure assessment when carried out by computational electromagnetics (mostly related to fixed installations).

This paper, first, briefly focuses on the current concepts for EMF exposure monitoring and associated research challenges. Second, it presents the status of monitoring activities in Europe. Third, options for personal EMF monitoring approaches will be described and evaluated against the background of existing concepts. Finally, we conclude with highlighting the relevance of personal exposure monitoring in light of technology dynamics, research needs and policy requirements. 

## 2. Concepts

In conceptual terms, we differentiate in this paper between emission, ambient exposure (sometimes also termed “immission”), personal exposure and dose monitoring [12]. Table 1 characterizes the concepts as well as the strengths and limitations of these different monitoring concepts. 

## 3. EMF monitoring activities in European Countries

### 3.1. Existing Reports

The EIS-EMF Project (European Information System on Electromagnetic Fields Exposure and Health Impacts) performed a general review of the exposure assessment activities [13]. In 2010 and 2014, reports by the European Health Risk Assessment Network on Electromagnetic Fields Exposure (EFHRAN), a project funded by the European Commission—Executive Agency for Health and Consumers (EAHC), were issued [5,14], in 2011 a French study summarized ongoing monitoring activities in Europe [15], and in 2012 an international survey on RF exposure was published [16]. The most comprehensive report, which will be discussed in this paper, was published in Switzerland [12]. 

**Table 1:** Key monitoring concepts.

Monitoring Concept	Characterisation
**Emission Monitoring**	
Monitoring of radiated power levels of infrastructure equipment and consumer devices. Used by regulators to control legislated/standardised maximum power output from single sources (devices or installations).	Emission monitoring primarily records the power (in Watt) or current (in Ampere) fed into a source, or measures—generally in close proximity to the source—the radiated electromagnetic field; *i.e.*, E (electric) fields and H (magnetic) fields. Well developed for fixed site installations. For devices worn or carried by a person it is restricted to worst case scenarios (not to actual emissions). No information about total ambient exposure levels (distribution, field strengths) or human exposure levels (incident field strengths, absorbed dose).
**Ambient Exposure Monitoring**	
Detection of indoor and/or outdoor field levels. Spatial resolution may vary from single spot data to rather comprehensive local or regional data sets produced by systematic measurement campaigns or by propagation modelling.	Ambient exposure monitoring records the downstream fields (E fields, H fields), *i.e.*, the fields in the wider environment of a source. At most places ambient exposures consist of more than just a single source. Exposure levels are measured either with broadband antennas, or summed up from frequency selective measurements, or they are calculated by simulation software. Allows detection of spatial and temporal trends. Outdoor data cannot be used to extrapolate to indoor data and vice versa. No information about personal or population exposure because human exposure depends on the time people spend in a specific environment and includes the exposure from close-to-body devices. These sources are generally not accounted for in ambient exposure monitoring campaigns.
**Personal Exposure Monitoring**	
Monitoring of incident field levels at the location of persons. Measurement duration ranges typically from a few hours to a maximum of one week. Measurement data may be complemented with activity diary and GPS data.	Personal exposure monitoring records the fields (E fields, H fields) at the location of the body, or very close to this location. Because people move, personal exposure monitoring requires mobile measurements with a portable device (exposimeter). This approach takes into consideration the behaviour of the people. All sources (fixed installations, mobile devices, indoor, outdoor) can be included. However, exposure from equipment used close to the body (electric appliances, DECT and mobile phones, other wireless consumer goods) cannot yet be reliably assessed. The statistical significance of personal exposure data strongly depends on the number of persons included into a measurement campaign.
**Dose Monitoring**	
Assessment of the in-body fields induced by personal exposure to external sources. Several dose metrics exist.	The electromagnetic dose is quantified in terms of electric or magnetic fields strengths or in terms of absorption of energy either per unit mass of tissues (the Specific Absorption Rate, SAR) or per unit area of exposed tissues (power density). In the absence of an established biomarker no in-situ measurements are possible. Dose assessment is based on comprehensive computer simulations. It is widely used for worst-case calculations in compliance testing. For monitoring purposes, dose monitoring is not feasible.

According to these documents, most national monitoring activity is oriented towards measurement campaigns. Modelling is rather exceptional. Monitoring of intermediate frequencies (IF) does not exist at all, and monitoring of extremely low frequency (ELF) fields is only exceptionally applied. The most common activity concerns ambient radio frequency (RF) field measurements in response to citizen requests, mostly in the context of newly erected mobile communication base-station antennas. 

The design of measurement campaigns in terms of number of sites and applied measurement protocols differs very much between the countries. This is all the more true for regional monitoring as implemented, for instance, in some German and some Swiss states. Several campaigns communicate the data on a web-based platform. 

With the notable exception of some epidemiological studies virtually nothing exists on the level of personal exposure monitoring. One reason for that is the fact that averaging of RF exposure signals is complex and that a large proportion of collected data is generally below the detection limits of available measurement equipment [17].

### 3.2. Survey

In the context of a feasibility study on EMF-monitoring options for Switzerland, a small survey about the state of monitoring activities in Europe was performed in fall 2011. Questionnaires (Excel sheets) were e-mailed to the country representatives of the COST Action BM0704. 

The following countries replied to the questionnaire: Austria, Bulgaria, Croatia, Cyprus, Denmark, Finland, France, Germany, Greece, Hungary, Ireland, Malta, Norway, The Netherlands, Portugal, Romania, Slovenia, Slovakia, Spain, Sweden, Switzerland, United Kingdom. For Italy, an important country in the context of EMF-monitoring, the relevant information was retrieved from published documents [14,15]. This data was included into the survey-results. The received Excel-sheets were analyzed manually.If answers were hard to interpret, the respondents were contacted and asked for short clarifications. 

We present the findings separately for measurement activities and for modelling/calculation exercises. For both activities, results are broken down into the ranges ELF (electricity), broadcasting services and mobile communication services. All responses have been categorized into: no activity, ad-hoc activity small/limited, *ad-hoc* activity large, systematic activity small/limited, systematic activity large, full inventory, no response/other.

The findings (Table 2 and Table 3) probably represent the most complete and most up-to-date picture about EMF monitoring activities in Europe available today. Table 2 summarizes the findings, Table 3 gives the necessary background information to the summary table. The overall picture looks as follows:

- EMF-monitoring activities are quite common and widely applied in Europe.
- Scale and scope of the activities are very diverse (absence of any common framework/paradigm).
- Most activity is oriented towards measurement campaigns. Modelling is rather exceptional.
- Monitoring of ELF fields does almost not exist.
- The most frequent activity concerns field measurements in response to citizen requests, mostly in the context of newly erected base-station antennas.
- The design of measurement campaigns in terms of number of sites and applied measurement protocol differs very much between the countries.
- Several “systematic” measurement campaigns (including web-based communication of the data) exist in Europe. In some countries (e.g., France), citizen requests led to the collection of a large amount of measurement data that is analysed as a whole every few years.

As a consequence little is known about the real exposure distribution in the population. New avenues in exposure monitoring would be needed as a countermeasure.

**Table 2:** Overview of country activities.

Country	Measurements			Modelling/Calculations		
	Radio/TV	Mobile	ELF	Radio/TV	Mobile	ELF
**Austria**						
**Bulgaria**						
**Cyprus**						
**Denmark**						
**Germany**						
**Spain**						
**Finland**						
**France**						
**Greece**						
**Hungary**						
**Ireland**						
**Italy**						
**Croatia**						
**Malta**						
**Netherlands**						
**Norway**						
**Portugal**						
**Romania**						
**Sweden**						
**Slovakia**						
**Slovenia**						
**Switzerland**						
**U.K.**						

Notes: brown color indicates “yearly, full inventory”; orange color indicates “yearly, large sample”; yellow color indicates “yearly, small sample”; dark green color indicates “*ad hoc*, many”; light green color indicates “*ad hoc*, few”; blue color indicates “no monitoring”; grey color indicates “not specified/other”.

**Table 3:** Specification of country activities.

Country	Radio/TV	Mobile Communication Networks	ELF
**Austria**	*ad hoc*, and workplace conformity check by AUVA in case of suspected problems with limits	*ad hoc*, and workplace conformity check by AUVA in case of suspected problems with limits	*ad hoc*, and workplace conformity check by AUVA in case of suspected problems with limits
**Bulgaria**	only when antenna characteristics change	only when antenna characteristics change	measurements when antenna characteristics change
**Cyprus**	all sites every 6 months	all sites every 6 months	measurements at about 10,000 locations
**Denmark**	no activities whatsoever	no activities whatsoever	no activities whatsoever
**Germany**	yearly measurements, sample size 2000 (Radio/TV/Mobile) selected by chance, total immission		no monitoring
**Spain**	yearly measurements, sample size 150, various selection criteria, changing sites, total RF immission		new infrastructure; measurement protocol not specified
**Finland**	*ad hoc*	*ad hoc*	*ad hoc*
**France**	about 2500 measurements p.a. at hot spots, mostly requested by citizens, mostly mobile basestations. 2007 last synthesis report. No differentiation between broadcasting and mobile communication		*ad hoc* measurements
**Greece**	*ad hoc*	20% of all sites selected by chance	*ad hoc*
**Hungary**	sample of 5 installations, yearly measurements and calculations	sample of 60 installations (yearly measurements), 25 installations selected for calculations	sample of 5 sites for yearly measurements
**Ireland**	since 2003, measurements at 900 installations (mainly base stations). At present, roughly 20–30 measurements p.a. Frequency selective peak measurements, no calculations		*ad hoc*
**Italy**	yearly measurements (various and variable) at several hundred installations (mainly base stations), broadband measurements, no differentiation between broadcasting and mobile communication		measurements in Torino (2006–2008)
**Croatia**	yearly ±10% of all installations (measurements and calculations)	yearly ±10% of all installations (measurements and calculations)	not specified
**Malta**	yearly, all installations (20)	yearly, all installations (500)	not specified
**Netherlands**	measurements: yearly, all installations, and ad hoc on public request; *ad hoc* calculations		*ad hoc* measurements
**Norway**	*ad hoc*	*ad hoc*	*ad hoc*
**Portugal**	*ad hoc* (about 100 measurements p.a., no differentiation between broadcasting and mobile communication		not specified
**Romania**	*ad hoc* on request, about 20 p.a.	*ad hoc* on request, about 100 p.a.	not specified
**Sweden**	no monitoring	10 sites permanent measurements, and 5 sites annually selected by chance. Calculations at selected hot spots	no monitoring
**Slovakia**	at least all 3 years measurements at all installation sites	at least all 3 years measurements at all installation sites	ad hoc measurements and calculations
**Slovenia**	yearly monitoring measurements at a few dozen installations	yearly monitoring measurements at a few dozen installations	yearly monitoring measurements at a few dozen installations
**Switzerland**	Calculations and measurements at new installations	Calculations and measurements at new installations, ad hoc measurements at selected locations, emission monitoring (24 h data for all sites), systematic ambient exposure monitoring in central Switzerland (measurements and calculations)	Calculations and measurements at new installations
**UK**	no measurements	ad hoc measurements on request, roughly 50 sites per year	a few *ad hoc* measurements on request

Note: p.a. = per annum

## 4. Moving from Ambient to Personal Exposure Monitoring

The major current monitoring deficiency concerns personal exposure. Ambient data as well as compliance data do not allow any firm conclusions about levels of personal or population exposure. Reasons therefore are, among others, first: ambient data are not informative for assessing exposure of people when the trajectories of movements are unknown. Second, the resolution of ambient data is often low, especially in the vertical dimension, or very uncertain, for instance regarding absorption and scattering by environmental structures. Third, worst case data from compliance measurements do not inform about the power emitted by close-to-body devices in daily use, e.g. mobile phones and tablets. Personal exposure assessment relating to such devices requires specific equipment and software [18,19] and is still a research challenge.

In the last few years, this deficiency has been addressed in research. Most past studies have focused on personal exposure induced by infrastructures like base stations or high voltage power lines or has considered separately the exposure from infrastructure (e.g., base stations) and those from devices used close to the body (e.g., mobile phones). The real exposure is in fact induced by both sources (in the case of mobile communication: the up- and down-link together, for an example see [20]). Several studies in the radiofrequency (RF) domain have demonstrated that exposure induced by devices used close to the body is clearly higher compared to exposure from far-field sources, *i.e.*, mobile networks, broadcasting or WLAN antennas [19,21,22,23,24,25]. As noted earlier, however, no exposure assessment paradigm for close-to-body sources that meets monitoring requirements is available to date. A key objective of current research is therefore to develop monitoring tools for all types of human exposure.

In the following sub-section we will list and discuss the most common exposure monitoring options, including gaps and limitations regarding personal exposure assessment. We will differentiate between options for ambient, personal and close-to-body monitoring approaches (see Table 4), with latter still lacking any implementable methodology.

**Table 4:** Exposure monitoring approaches.

Approach	Section in the Paper	Exposure to Installations	Exposure to Close-to-Body Devices
**Ambient Exposure Monitoring**			
Fixed Site Transmitter Modelling	4.2	Outdoor	No
High Spatial Resolution Modelling	4.3	Outdoor, indoor	From third parties’ devices
**Personal Exposure Monitoring**			
Representative Sample with Exposimeters	4.4	Outdoor, indoor	From third parties’ devices
Quota Sample with Exposimeters	4.4	Outdoor, indoor	From third parties’ devices
**Close-to-body Exposure Monitoring**			
Emission Monioring	4.1	No	From own devices
Exposure Measurements	4.5	No	From own devices

### 4.1. Emission Monitoring

So far, most emission monitoring has focused on fixed site transmitters such as mobile phone base stations or broadcast transmitters. However, for personal exposure monitoring a better understanding of the emissions from sources close-to body in daily life is needed. Currently, very little is known about the typical output power of mobile phones in a network and even less when being in stand-by mode. Output power in stand-by mode is expected to be heavily affected by many factors such as the type of phone, the configuration of the network, the number of “apps” installed on smart phones, the behavior of the person (travelling, being inside, outside) *etc.* [25]. Without such knowledge, dose estimation cannot be done for real life scenarios.

A main challenge towards this objective is to substitute the current exposure assessment methods for close-to-body sources, based on worst-case scenarios [26,27], with methods and equipment able to quantify levels of daily use [28]. The FP7 EU LEXNET project [29] has started to work to define an exposure index for selected RF exposures that will aggregate the downlink exposure caused by mobile phone base stations, the uplink exposure caused by the devices in communication, the different usage patterns, the category of users, the user posture and device position, the different environments, the different radio access technologies and layers in the network. 

Further, the FP7 EU SEAWIND project [30] provided a comprehensive assessment of the incident field exposure of installed wireless local area networks (WLAN or WiFi) or wireless metropolitan area networks (WMAN or WiMAX), body-mounted and body-worn wireless personal area networks (WPAN) and WLAN devices. Using high-resolution anatomically MRI-based surface models that represent a wide spectrum of the human population, the induced fields in the human body will be numerically determined. 

### 4.2. Fixed Site Transmitter Modelling

By means of propagation models, the spatial distribution of average or peak field strengths, from mainly large infrastructures, is calculated and mapped [31,32,33,34,35,36,37,38,39]. Radio engineers, for instance, use such software for radio planning purposes. In the radiofrequency domain, simulation software generally calculates ambient electric field strengths. The uncertainty of such calculations depends on the quality of the input data such as the antennae characteristics, building and topographic data. The application of GISMap software in Switzerland, for instance, resulted in a general uncertainty in the order of magnitude of ±50% (3–4 dB) for total field strength (data derived from short term measurements representative for average daytime conditions [32]). This uncertainty may increase with a focus on single services or with a reduction of average times; it may decrease with longer average times and with averaged validation data (instead of spot measurement data). In a sample of 164 volunteers Spearman rank correlation between mean personal mobile phone base station exposure during one week at all places where people stayed and modeled exposure at home was 0.71 (95%-CI: 0.63 to 0.78) [40]. Similar thinking applies to ELF exposure. However, variability is somewhat less accentuated and the simulated H-field strengths distribution in space is much more robust compared to radiofrequency fields. However, the exposure patterns are very local with significant field strengths (>0.4 µT) in the very close vicinity (±200 m) of power lines only [41].

### 4.3. High Spatial Resolution Monitoring

In ambient measurement campaigns, exposures at defined locations are recorded [34,37,42,43,44,45,46]. The measurement may be a spot, a short-time (from a few hours to a few days), a long-term (several weeks or months), or a periodic measurement (e.g., periodic short-time measurements). Locations for the probes may be selected by random or by systematic sampling. Generally, outdoor locations are selected, however, indoor levels may also be monitored [47,48,49]. In case of long-term or periodic measurements at different locations, the time-series data allow to identify exposure trends [50]. Depending on the detected frequencies, such data cover selected frequency bands only or the whole spectrum, *i.e.*, they show trends in background radiation. 

Recently ambient field levels have been recorded on pre-defined measurement trajectories in selected compartments (microenvironments) using portable measurement [51]. A compartment is defined as a locality which matters in terms of daily human behavior. Examples of compartments are indoor environments like households, workplaces, shopping centers, *etc.*, outdoor environments like inner cities, rural recreational areas, suburbs, villages, *etc.*, and mobile environments like commuting by car, train, bus, or long-distance travelling by car or train. 

A campaign may look like this: ten types of compartments (e.g., residential areas, downtown, trains, railway station, shopping centers, *etc.*) will be defined and about 5–10 specific compartments per type will be selected. Measurements will be done on two different measurement trajectories in each compartment. The measurements are performed twice at different time slots and repeated 3–4 times. A time slot may cover 10–30 min. The whole campaign can be scheduled once or repeated several times, e.g., every year, depending on budgetary and statistical requirements.

This monitoring approach allows, first, to identify typical (not: statistically representative) exposure levels in interested compartments, second, to record overall and compartment specific exposure trends, third to construct personal exposure profiles based on lifestyles. A lifestyle can be defined with the help of the number of times a person spends in specific compartments. Personal exposure can then roughly be assessed for such ideal lifestyles. The approach can also be combined with exposure modelling [40,52], although exposure from very small installations that do not need an authorization (e.g., femto cells) can only be included in the measurements but not in the modelling due to lack of input data. Exposure to fields from electric and wireless appliances used by third parties in the vicinity is included. However, exposure to devices worn or carried by persons has to be assessed separately [53] (see also section 4.4).

In statistical terms, data variability strongly depends on the number of measurement series performed. In general, the data does not adequately account for daily variations in field levels but is able to capture long term trends. Weekly variations may be slightly better represented. Because the equipment is handled and the measurements are performed by professional personnel, following a defined protocol, the data is reliable and credible. 

Another possibility of ambient exposure monitoring is mobile probing [54]. Measurement equipment is mounted on vehicles, for instance buses, tramways, cabs, rental cars, cars of a business fleet, *etc.* Both measurement locations (with the help of GPS data), and measurement times are logged. This allows covering a larger area and if the data is “thick” enough, it allows mapping ambient exposures over time. However, the approach is not suitable for indoor sources and—depending on the vehicles used—limited to locations with high population density (e.g., cities) and/or public transportation coverage. Additionally, the exposure contribution from other people’s mobile phones will be underestimated, as the distance to the people will be larger than for a person carrying a mobile device.

### 4.4. Personal Monitoring

The most comprehensive personal exposure monitoring approach consists in selecting a representative population sample that records for several days personal exposure with the help of an exposimeter, as in [55,56] and in an exploratory spirit or for feasibility purposes in [57,58,59,60,61,62,63,64,65,66,67]. However, costs for such a campaign are quite high. Representativity of the study sample is difficult to ensure, since such measurements are demanding for the volunteers and participation rate may be low. Thus, selection bias is of concern. An alternative is to select subjects from interested lifestyle groups (quota sampling). 

A personal measurement campaign may look like this: Definition of, for instance, six lifestyle groups (young urban employee, older urban employee, young rural employee, older rural employee, older non-employed person, pupil/student). In case of 20 subjects per group and 48-hour measurements during one week, 240 days of data will be recorded. If six measurement devices are available, the campaign can be performed in roughly three to four months. Depending on budgetary and statistical requirements, the sample size could be increased or the measurements could be repeated. 

Such data does not allow generalizations to the population at large. Nevertheless, it informs about typical exposure levels and patterns, about exposure differences between lifestyle groups, and about exposure trends. Yet, interpretation of the data is challenging. A diary that logs the activities of the subjects and links this information with the exposure data strongly supports data analysis and interpretation. It allows, for instance, to identify and compare exposures in/between different compartments (microenvironments)—indoor, outdoor, on the move—and for different activities. The latter is especially important for assessing the contribution of exposure from devices used close to the body to overall personal exposure. However, it has to be noted that presently, no methodology exists to readily account for this contribution. Such methodology had to meet, among others, the following challenges associated with the correction of measurement data: (i) accounting for the distance between consumer device (source) and exposimeter, (ii) respecting the variability of this distance due to changes in device handling, (iii) incorporating the shielding effects of the body. It has been suggested that personal distributed exposure meter may be a solution to deal with this problem [68]. 

The validity of the data is lower compared to measurements in compartments because the equipment is handled by laypersons. Whether the measurement protocols are followed by the subjects cannot be easily verified. 

Another issue is the statistical precision. In several studies personal exposure data was analyzed with regard to its statistical characteristics [69,70,71,72,73]. In the QUALIFEX project, for instance, 160 subjects were equipped with a personal exposimeter carried during one week. The estimated uncertainty (expressed as the 95% confidence interval of the estimated mean) for 100 weekly measurements ranged from ±10% for total exposure, up to ±50% for some specific frequency bands. The data validity from smaller samples can be increased by longer measurement periods, although the sample size seems to be more crucial than the measurement period. 

A future alternative to personal measurements may be crowd sensing, *i.e.*, EMF recording with modified and/or expanded smartphones used by large populations. This approach may become feasible at a later stage when enough experiences with personal exposure meters have been collected [74].

### 4.5. Dose Modelling, Gaps and Open Issues

To finally model and monitor the EMF dose of the population, the above mentioned components have to be integrated since direct dose monitoring is very difficult to conduct. First, direct dose measurements cannot be performed in the absence of an established biomarker. Second, simulations are subject to significant uncertainties stemming from, among others, complexities of the anatomy of the human body, uncertainties of tissue parameters, arbitrary choices about the modelling framework, computer power constraints, and, probably most important, uncertainties about the exposure source data. In the case of sources used close to the body the uncertainties are even more pronounced because of large variations in the individual device handlings und usage patterns and of the generally huge variety of models and technical characteristics of the devices. All these factors contribute strongly to the dose. 

In order to estimate real life doses [28], source modelling and measurement data will be combined with detailed digital human models [75], derived from MRI-scans. In this way not only whole-body doses, but also organ and tissue specific local doses (e.g., mobile phone radiation, exposures by electric household appliances) [26,28,76,77,78] can be estimated. Depending on the health outcome of interest, different organ or tissue exposures may be relevant. However, this dose modelling is faced with a series of challenges that have not yet been sufficiently investigated and where only limited experiences exist [79]:

- Near-field (close-to-body) sources: exposure from portable consumer goods (mobile phones, DECT phones, Bluetooth and WiFi equipment, to list but devices from the RF domain) represent a major, or even—especially for young people being heavy users of these commodities—the dominant, source of personal exposure [69]. Better knowledge about the emission patterns of these sources is needed as mentioned in section 4.1., and has to be combined with not yet existing data on detailed usage behavior (e.g., duration and posture of use). Potentially, crowd sensing approaches may also be useful for gaining such data.
- Uncertainty assessment: which uncertainty budgets have to be taken into account due to emission variability of the devices, due to the variability in handling devices (frequency, duration and practice), and due to the variability in the location of measurement antennas?
- Measurement accuracy: what is the uncertainty of personal measurement devices, in particular regarding crosstalk between adjacent frequency bands, harmonics, or lack of frequency bands in many current devices [79,80,81,82,83,84]? Also the impact of body shielding on the measurements has to be considered [85,86,87]. Recently, an approach using body worn antennas—e.g., integrated into textiles for a distributed personal exposimeter [68]—has been proposed to address this problem.
- Reference volume: what is a biologically sensible and technically feasible reference volume and what measurement locations (single point, multiple points) have to be selected to realistically cover the defined volume for personal exposure assessment of far-field sources?
- Exposure metrics: no scientifically convincing personal exposure metrics for monitoring purposes have been established so far. The basic quantity metrics inside the body relates to induced biological effects, *i.e.*, nerve stimulation and heating. These well-established biological effects are controlled, for instance, in the ICNIRP guidelines by the basic restrictions [6,7]. However, endpoints relating to potential non-thermal effects require an exposure metric that takes signal forms and strengths into account [88]. Such exposure information collected in the context of monitoring campaigns and/or epidemiological research would have considerable practical relevance. For instance, the explanatory power of future prospective cohort studies strongly depends on an exposure metric comprehensive enough to address several potential health endpoints.

Against the background above, Table 5 summarises the significance and limitations of the three discussed options of personal (population) exposure assessment, *i.e.*, modelling exposure by means of high spatial resolution monitoring, measuring exposure by means of exposimeters in a representative sample, measuring exposure by means of exposimeters in a quota sample. In all three approaches, the assessment of exposure by the (own) use of devices used close to the body still needs to be resolved.

**Table 5:** Options for Personal Exposure Monitoring.

Approach	Selection Criteria	Significance	Limitations
High Spatial Resolution Monitoring	Different types of compartments (microenvironments)	Quick collection of highly reproducible measurements for a wider range of compartments	Representativiy of the measurements for larger areas, no account for exposure to own use of close-to-body devices
Representative Sample with Exposimeters	Random or convenient population sample	Data for real exposure of population	Limited reliability of data gathering, no account for exposure to own use of close-to-body devices, very expensive, possible bias in volunteer selection
Quota Sample with Exposimeters	Life-style groups	Data for real exposure of selected sub-populations (real types)	Limited reliability of data gathering, no account for exposure to own use of close-to-body devices, very expensive

## 5. Conclusions

As a key challenge for future EMF monitoring we recognize the need to change from ambient to personal exposure assessments and eventually to estimate dose for corresponding monitoring. The drivers behind this need are both technological developments, and increased scientific insights into biological and health related effects of EMF exposures. The RF studies that have been performed previously have mainly considered infrastructure or mobile devices separately and therefore do not provide a clear view of the real personal exposure induced by wireless communication systems. Furthermore, it is expected that the complexity of EMF exposures continues to increase. This is underlined by the fact that according to EU [89] the worldwide mobile traffic alone will be 33 times higher by 2030 compared to 2010 figures. To enable such an increase, the future communication networks will involve, to name but two, more powerful provider infrastructures or/and mobile data offloading, *i.e.* the use of complementary small cell technologies like femtocells or WiFi for delivering data originally targeted for 3G/4G networks. In addition, the current technology development in the electricity sector towards smart grids will very likely involve new exposure patterns in the context of smart home technologies and electric vehicles. All these developments will make exposure assessment and monitoring both complex and inevitably necessary.

The complexity of the assessment and monitoring task is also illustrated by the foreseen expansion into the IF and TF bands in the near future. An increasing number of devices and processes employing these frequency domains (household appliances, security devices, telecommunication *etc.*) will be/are already introduced into everyday life. Almost nothing is known about these exposures and potential exposure levels.

We identified as a major challenge in the shift from ambient to personal exposure monitoring the development and implementation of appropriate measurement equipment and methods, and of monitoring campaigns. Current equipment used to assess EMF exposure has a series of deficiencies for estimating the real exposure of a person or the population, and / or to reliably monitor personal exposure. A key deficiency concerns the assessment of exposure from devices used close to the body. The contribution of this exposure to total personal exposure is significant and cannot be neglected.

In light of these shortcomings and in face of the pressing need to monitor personal EMF exposure for both health and policy purposes, we discussed two options for assessing human exposure: first, high resolution measurements of ambient exposure levels in selected compartments (microenvironments) relevant for daily life; second, personal exposure measurements with portable devices. Our suggestions are preliminary and have to be further investigated. Epidemiology is currently the main driver for equipment innovations, and for the paradigm shift from ambient to personal exposure assessment.

Any sustainable exposure policy relies on public support and acceptance. Without such backing, it will face citizen and/or local authority opposition, at least in democratic countries. As the EUROBAROMETER data show, the EMF topic is characterised by general concerns, and partly inappropriate and volatile perceptions. Without robust data about the real exposure of people, policy decisions and legislations are hard to “sell”, and science and risk communication is prone to fail. In this view, the development and implementation of a new EMF monitoring paradigm and approach oriented towards personal exposure is a necessary step for both an evidence-based exposure policy and a pro-active communication about human EMF exposure. 

## References

[1] ICNIRP (International Commission on Non-Ionizing Radiation Protection) (2008). Statement on EMF-emitting new technologies. Health Phys..

[2] SCENHIR (Scientific Committee on Emerging and Newly Identified Health Risks) (2009). Health Effects of Exposure to EMF.

[3] IARC (International Agency for Research on Cancer) (2002). Non-Ionizing Radiation, Part 1: Static and Extremely Low-Frequency (ELF) Electric and Magnetic Fields.

[4] IARC (International Agency for Research on Cancer) (2013). Non-Ionizing Radiation, Part 2: Radiofrequency Electromagnetic Fields.

[5] Grellier J., Ravazzani P., Cardis E. (2014). Potential health impacts of residential exposures to extremely low frequency magnetic fields in Europe. Environ. Int..

[6] ICNIRP (International Commission on Non-Ionizing Radiation Protection) (2010). Guidelines for limiting exposure to time-varying electric, magnetic fields (1 Hz–100 kHz). Health Phys..

[7] ICNIRP (International Commission on Non-Ionizing Radiation Protection) (1998). Guidelines for limiting exposure to time-varying electric, magnetic, and electromagnetic fields (up to 300 GHz). Health Phys..

[8] IEEE/ICES (International Committee on Electromagnetic Safety) (2006). IEEE Standard for Safety Levels with Respect to Human Exposure to Radio Frequency Electromagnetic Fields, 3 kHz to 300 GHz. Standard C95.1–2005.

[9] EU (European Union) (1999). Council Recommendation of 12 July 1999 on the Limitation of Exposure of the General Public to Electromagnetic Fields (0 Hz to 300 GHz).

[10] EU (European Union) (1999). Directive 1999/5/ of the European Parliament and of the Council of 9 March 1999 on Radio Equipment and Telecommunications Terminal Equipment and the Mutual Recognition of Their Conformity.

[11] Eurobarometer (2010). Eurobarometer 347, Wave 73.3, Electromagnetic Fields.

[12] Dürrenberger G., Bürgi A., Fröhlich J., Kuster N., Röösli M. (2012). NIS-Monitoring Schweiz.

[13] EIS-EMF (2005). European Information System on Electromagnetic Fields Exposure and Health Impacts.

[14] Thuroczy G., Gajsek P., Samaras T., Wiart J. (2010). Report on the Level of Exposure (Frequency, Patterns and Modulation) in the European Union.

[15] BIO Intelligence Service (2011). Revue Internationale de L’organisation et de la Stratégie de Surveillance des Ondes Electromagnétiques.

[16] Rowley J.T., Joyner K.H. (2012). Comparative international analysis of radioafrequency exposure surveys of mobile communication base stations. J. Expo. Sci. Environ. Epidemiol..

[17] Gajsek P., Ravazzani P., Wiart J., Grelier J., Samaras T., Thuroczy G. (2013). Electromagnetic field (EMF) exposure assessment in Europe—Radio frequency fields (10 MHz–6 GHz). J. Expo. Sci. Environ. Epidemiol..

[18] Gati A., Hadjem A., Wong M.F., Wiart J. (2009). Exposure induced by WCDMA Mobiles Phones in operating networks. IEEE Trans. Wirel. Commun..

[19] Cardis E., Deltour I., Mann S., Moissonnier M., Taki M., Varsier N., Wake K., Wiart J. (2008). Distribution of RF energy emitted by mobile phones in anatomical structures of the brain. Phys. Med. Biol..

[20] Boursianis A., Vanias P., Samaras T. (2012). Measurements for assessing the exposure from 3G femtocells. Radiat. Prot. Dosim..

[21] Neubauer G., Feychting M., Hamnerius Y., Kheifets L., Kuster N., Ruiz I., Schüz J., Uberbacher R., Wiart J., Röösli M. (2007). Feasibility of future epidemiological studies on possible health effects of mobile phone base stations. Bioelectromagnetics.

[22] Wiart J., Hadje A., Varsier N., Bloch I., Person C.H., Gati A., Conil E. Comparative Analysis of the Human Exposure induced by RF Mobile Phones and Base Stations. Proceedings of the URSI International Symposium on Electromagnetic Theory EMTS.

[23] Wiart J., Dale C., Bosisio A.V., le Cornec A. (2000). Analysis of the influence of the power control and discontinuous transmission on RF exposure with GSM mobile phones. IEEE Trans. Electromagn. Compat..

[24] Mohler E., Frei P., Aydin A., Bürgi A., Röösli M. (2009). Personal exposure to high-frequency electromagnetic fields in the region of Basel. An overview of the QUALIFEX study. Umwelmedizin in Forschung und Praxis.

[25] Urbinello D., Röösli M. (2013). Impact of one’s own mobile phone in stand-by mode on personal radiofrequency electromagnetic field exposure. J. Expo. Sci. Environ. Epidemiol..

[26] Kühn S., Lott U., Kramer A., Kuster N. (2007). Assessment methods for demonstrating compliance with safety limits of wireless devices used in home and office environments. IEEE Trans. Electromagn. Compat..

[27] Kühn S. (2009). EMF Risk Assessment: Exposure Assessment and Compliance Testing in Complex Environments. Ph.D. Thesis.

[28] Lauer O., Frei P., Gosselin M.C., Joseph W., Röösli M., Fröhlich J. (2013). Combining near- and far-field exposure for an organ-specific and whole-body RF-EMF proxy for epidemiological research: A reference case. Bioelectromagnetics.

[29] Low EMF Exposure Networks (LEXNET) Grant Agreement No. 318273. http://www.lexnet-project.eu.

[30] Sound Exposure and Risk Assessment of Wireless Network Devices (SEAWIND) Grant Agreement No.244149. http://seawind-fp7.eu.

[31] Bürgi A. (2011). Immissionskataster für Niederfrequente Magnetfelder von Hochspannungsleitungen—Machbarkeits- und Pilotstudie.

[32] Bürgi A., Frei P., Theis G., Mohler E., Braun-Fahrländer C., Fröhlich J., Neubauer G., Egger M., Röösli M. (2010). A model for radiofrequency electromagnetic field predictions at outdoor and indoor locations in the context of epidemiological research. Bioelectromagnetics.

[33] Bürgi A., Theis G., Siegenthaler A., Röösli M. (2008). Exposure modelling of high frequency electromagnetic fields. J. Expo. Sci. Environ. Epidemiol..

[34] Bornkessel C., Schubert M., Wuschek M., Schmidt P. (2007). Determination of the general public exposure around GSM and UMTS base stations. Radiat. Prot. Dosim..

[35] Beekhuizen J., Vermeulen R., Kromhout H., Bürgi A., Huss A. (2013). Geospatial modelling of electromagnetic fields from mobile phone base stations. Sci. Total Environ..

[36] Aerts S., Deschrijver D., Verloock L., Dhaene T., Martens L., Joseph W. (2013). Assessment of outdoor radiofrequency electromagnetic field exposure through hotspot localization using kriging-based sequential sampling. Environ. Res..

[37] Aerts S., Deschrijver D., Joseph W., Verloock L., Goeminne F., Martens L., Dhaene T. (2013). Exposure assessment of mobile phone basestation radiation in an outdoor environment using sequential surrogate modeling. Bioelectromagnetics.

[38] Kim B.C., Park S.O. (2010). Evaluation of RF electromagnetic field exposure levels from cellular base stations in Korea. Bioelectromagnetics.

[39] Joseph W., Verloock L., Goeminne F., Vermeeren G., Martens L. (2012). Assessment of RF exposure from emerging wireless technologies in different environments. Health Phys..

[40] Frei P., Mohler E., Bürgi A., Fröhlich J., Neubauer G., Braun-Fahrländer C., Röösli M. (2010). Classification of personal exposure to radio frequency electromagnetic fields (RF-EMF) for epidemiological research: Evaluation of different exposure assessment methods. Environ. Int..

[41] Huss A., Spoerri A., Egger M., Röösli M. (2009). Residence near power lines and mortality from neurodegenerative diseases: Longitudinal study of the Swiss population. Amer. J. Epidemiol..

[42] Henderson S.I., Bangay M.J. (2006). Survey of RF exposure levels from mobile telephone base stations in Australia. Bioelectromagnetics.

[43] Line P., Cornelius W.A., Bangay M.J., Grollo M. (2000). Levels of Radiofrequency Radiation from GSM Mobile Phone Base Stations.

[44] Rufo M.M., Paniagua J.M., Jimenez A., Antolín A. (2011). Exposure to highfrequency electromagnetic fields (100 KHz–2 GHz) in Extremadura (Spain). Health Phys..

[45] Troisi F., Boumis M., Grazioso P. (2008). The Italian national electromagnetic field monitoring network. Ann. Telecommun..

[46] Stratmann M., Wernu C. (1994). Exposure of the Swiss Population to 50 Hz Magnetic Fields: PSI Annual Report 1994.

[47] Neitzke H.P., Osterhoff J., Peklo K., Voigt H. (2007). Determination of exposure due to mobile phone base stations in an epidemiological study. Radiat. Prot. Dosim..

[48] Tomitsch J., Dechant E., Frank W. (2010). Survey of electromagnetic field exposure in bedrooms of residences in lower Austria. Bioelectromagnetics.

[49] Röösli M., Jenni D., Kheifets L., Mezei G. (2011). Extremely low frequency magnetic field measurements in buildings with transformer stations in Switzerland. Sci. Total Environ..

[50] Tomitsch J., Dechant E. (2011). Trends in residential exposure to electromagnetic fields from 2006 to 2009. Radiat. Prot. Dosim..

[51] Urbinello D., Huss A., Beekhuizen J., Vermeulen R., Röösli M. (2014). Use of portable exposure meters for comparing mobile phone base station radiation in different types of areas in the cities of Basel and Amsterdam. Sci. Total Environ..

[52] Frei P., Mohler E., Bürgi A., Fröhlich J., Neubauer G., Braun-Fahrländer C., Röösli M. (2009). A prediction model for personal radio frequency electromagnetic field exposure. Sci. Total Environ..

[53] Kelsh M.A., Shum M., Sheppard A.R., Mcneely M., Kuster N., Lau E., Weidling R., Fordyce R., Kühn S., Sulser C.H. (2011). Measured radiofrequency exposure during various mobile-phone use scenarios. J. Expo. Sci. Environ. Epidemiol..

[54] Estenberg J., Augustsson T. (2014). Extensive frequency selective measurements of radiofrequency fields in outdoor environments performed with a novel mobile monitoring system. Bioelectromagnetics.

[55] Thomas S., Kühnlein A., Heinrich S., Praml G., Nowak D., von Kries R., Radon K. (2008). Personal exposure to mobile phone frequencies and well-being in adults: A cross-sectional study based on dosimetry. Bioelectromagnetics.

[56] Thomas S., Heinrich S., von Kries R., Radon K. (2010). Exposure to radio-frequency electromagnetic fields and behavioural problems in Bavarian children and adolescents. Eur. J. Epidemiol..

[57] Viel J.-F., Clerc S., Barrera C., Rymzhanova R., Moissonnier M., Hours M., Cardis E. (2009). Residential exposure to radiofrequency fields from mobile-phone base stations, and broadcast transmitters: A population-based survey with personal meter. Occup. Environ. Med..

[58] Viel J.F., Cardis E., Moissonnier M., de Seze R., Hours M. (2009). Radiofrequency exposure in the French general population: Band, time, location and activity variability. Environ. Int..

[59] Thuróczy G., Molnár F., Jánossy G., Nagy N., Kubinyi G., Bakos J., Szabó J. (2008). Personal RF exposimetry in urban area. Ann. Telecommun..

[60] Radon K., Spegel H., Meyer N., Klein J., Brix J., Wiedenhofer A., Eder H., Praml G., Schulze A., Ehrenstein V. (2006). Personal dosimetry of exposure to mobile telephone base stations? An epidemiological feasibility study comparing the Maschek dosimeter prototype and the Antennessa DSP-090 system. Bioelectromagnetics.

[61] Joseph W., Frei P., Roösli M., Thuróczy G., Gajsek P., Trcek T., Bolte J., Vermeeren G., Mohler E., Juhász P. (2010). Comparison of personal radio frequency electromagnetic field exposure in different urban areas across Europe. Environ. Res..

[62] Joseph W., Frei P., Röösli M., Vermeeren G., Bolte J., Thuróczy G., Gajšek P., Trček T., Mohler E., Juhász P. (2012). Between-country comparison of whole-body SAR from personal exposure data in urban areas. Bioelectromagnetics.

[63] Frei P., Mohler E., Braun-Fahrländer C., Fröhlich J., Neubauer G., Röösli M. (2012). Cohort study on the effects of everyday life radio frequency electromagnetic field exposure on non-specific symptoms and tinnitus. Environ. Int..

[64] Röösli M., Frei P., Bolte J., Neubauer G., Cardis E., Feychting M., Gajsek P., Heinrich S., Joseph W., Mann S. (2010). Conduct of a personal radiofrequency electromagnetic field measurement study: Proposed study protocol. Environ. Health.

[65] Bolte J., Pruppers M., Kramer J., van der Zande G., Schipper C., Fleurke S. (2008). The Dutch exposimeter study: Developing an activity exposure matrix. Epidemiology.

[66] Joseph W., Vermeeren G., Verloock L., Heredia M., Martens L. (2008). Characterization of personal RF electromagnetic field exposure and actual absorption for the general public. Health Phys..

[67] Mann S.M., Addison D.S., Blackwell R.P., Khalid M. (2005). Personal Dosimetry of RF Radiation—Laboratory and Volunteers Trials of an RF Personal Exposuremeter.

[68] Thielens A., de Clercq H., Agneessens S., Lecoutere J., Verloock L., Declercq F., Vermeeren G., Tanghe E., Rogier H., Puers R. (2013). Personal distributed exposimeter for radio frequency exposure assessment in real environments. Bioelectromagnetics.

[69] Röösli M., Frei P., Mohler E., Braun-Fahrländer C., Bürgi A., Fröhlich J., Neubauer G., Theis G., Egger M. (2008). Statistical analysis of personal radiofrequency electromagnetic field measurements with nondetects. Bioelectromagnetics.

[70] Frei P., Mohler E., Neubauer G., Theis G., Bürgi A., Fröhlich J., Braun-Fahrländer C., Bolte J., Egger M., Röösli M. (2009). Temporal and spatial variability of personal exposure to radiofrequency electromagnetic fields. Environ. Res..

[71] Bolte J.F.B., Eikelboom T. (2012). Personal radiofrequency electromagnetic field measurements in the Netherlands: Exposure level and variability for everyday activities, times of day and types or area. Environ. Int..

[72] Bolte J., van der Zande G., Kamer J. (2011). Calibration and uncertainties in personal exposure measurements of radiofrequency electromagnetic fields. Bioelectromagnetics.

[73] Viel J.F., Tiv M., Moissonnier M., Cardis E., Hours M. (2011). Variability of radiofrequency exposure across days of the week: A population-based study. Environ. Res..

[74] Knafl U., Lehmann H., Riederer M. (2008). Electromagnetic field measurements using personal exposimeters. Bioelectromagnetics.

[75] Christ A., Kainz W., Hahn E.G., Honegger K., Zefferer M., Neufeld E., Rascher W., Janka R., Bautz W., Chen J. (2010). The Virtual Family—Development of surface-based anatomical models of two adults and two children for dosimetric simulations. Phys. Med. Biol..

[76] Kühn S., Cabot E., Christ A., Capstic M., Kuster N. (2009). Assessment of the radio-frequency electromagnetic fields induced in the human body from mobile phones used with hands-free kits. Phys. Med. Biol..

[77] Leitgeb N., Cech R., Schröttner J., Lehofer P., Schmidpeter U., Rampetsreiter M. (2008). Magnetic emissions of electric appliances. Int. J. Hyg. Environ. Health.

[78] Wiart J., Hadjem A., Varsier N., Conil E. (2011). Numerical dosimetry dedicated to children RF exposure. Prog. Biophys. Mol. Biol..

[79] Mann S. (2010). Assessing personal exposures to environmental radiofrequency electromagnetic fields. Comptes Rendus Phys..

[80] Blas J., Lago F.A., Fernández P., Lorenzo R.M., Abril E.J. (2007). Potential exposure assessment errors associated with body-worn RF dosimeters. Bioelectromagnetics.

[81] Iskra S., McKenzie R., Cosic I. (2010). Factors influencing uncertainty in measurement of electric fields close to the body in personal RF dosimetry. Radiat. Prot. Dosim..

[82] Lauer O., Neubauer G., Röösli M., Riederer M., Frei P., Mohler E., Fröhlich J. (2012). Measurement setup and protocol for characterizing and testing radio frequency personal exposure meters. Bioelectromagnetics.

[83] McDevitt J.J., Breysse P.N., Bowman J.D., Sassone D.M. (2002). Comparison of extremely low frequency (ELF) magnetic field personal exposure monitors. J. Expo. Anal. Environ. Epidemiol..

[84] Bornkessel C., Blettner M., Breckenkamp J., Berg-Beckhoff G. (2010). Quality control for exposure assessment in epidemiological studies. Radiat. Prot. Dosim..

[85] Neubauer G., Cecil S., Giczi W., Petric B., Preiner P., Fröhlich J., Röösli M. (2010). The association between exposure determined by radiofrequency personal exposimeters and human exposure: A simulation study. Bioelectromagnetics.

[86] Neubauer G., Cecil S., Giczi W., Petric B., Preiner P., Fröhlich J., Röösli M. (2008). Final Report on the Project C2006–07, Evaluation of the Correlation between RF Dosimeter Reading and Real Human Exposure.

[87] Bahillo A., Blas J., Fernández P., Lorenzo R.M., Mazuelas S., Abril E.J. (2008). E-field assessment errors associated with RF dosimeters for different angles of arrival. Radiat. Prot. Dosim..

[88] Foster K.R., Repacholi M. (2004). Biological effects of radiofrequency fields: Does modulation matter?. Radiat. Res..

[89] EC (European Commission) Mobile Communications: Fresh €50 Million EU Research Grants in 2013 to Develop “5G” Technology. https://www.metis2020.com/wp-content/uploads/2013/03/IP-13–159_EN.pdf.

